# Distinct modes of cell division drive *Anaplasma phagocytophilum* morphotype development and the infection cycle

**DOI:** 10.1128/mbio.01972-25

**Published:** 2025-08-25

**Authors:** Travis J. Chiarelli, Savannah E. Sanchez, Mary Clark H. Lind, Nathaniel S. O'Bier, Curtis B. Read, Richard T. Marconi, Jason A. Carlyon

**Affiliations:** 1 Department of Microbiology and Immunology, Virginia Commonwealth University Medical Center, School of Medicine, Richmond, Virginia, USA; Massachusetts Institute of Technology, Cambridge, Massachusetts, USA

**Keywords:** *Anaplasma phagocytophilum*, *Anaplasmataceae*, pleomorphic, cell division, morphotype, cell development, spore, cell differentiation, obligate intracellular bacterium

## Abstract

Pleomorphism is an evolutionary adaptation by which diverse microorganisms maximize their fitness by transitioning between morphologically distinct forms that perform disparate functions in response to the local microenvironment. Cell division is critical for morphotype transition in many pleomorphic bacterial systems. *Anaplasma phagocytophilum*, which causes the emerging disease granulocytic anaplasmosis, is a pleomorphic obligate intracellular bacterium that lives in a pathogen-modified vacuole except for when it is exocytically released for dissemination to naïve cells. This bacterium cycles between non-infectious, replicative reticulate cell (RC) and infectious, non-replicative dense-cored (DC) forms. Here, we establish that differential modes of *A. phagocytophilum* cell division drive morphotype development where RC bacteria divide symmetrically to expand the intravacuolar population after which they switch to sacrificial asymmetric division to produce DCs. *A. phagocytophilum* MreB is crucial for cell division, specifically septation, and thereby formation of both morphotypes. Inhibition of cell division prevents not only DC formation but also *A. phagocytophilum* vacuole maturation and infectious progeny release, which suggests that these pathogenic processes are coordinated. This study advances understanding of *A. phagocytophilum* growth and morphotype development and, thus, pathobiology. It also provides the first evidence linking cell division to morphotype development in the *Anaplasmataceae*.

**IMPORTANCE:**

*Anaplasma phagocytophilum*, an obligate intracellular bacterial pathogen that lives in a host cell-derived vacuole, causes human and veterinary diseases of global importance. In the pathogen-occupied vacuole, *A. phagocytophilum* transitions from a replicative, non-infectious morphotype to a non-replicative, infectious morphotype that is released to spread infection. We established that distinct modes of bacterial cell division drive not only *A. phagocytophilum* replication but also its differentiation to the infectious form and dissemination to naïve cells. How pleomorphism is regulated in most vacuole-adapted bacterial pathogens is poorly understood. Therefore, this study advances fundamental knowledge of vacuole-adapted pleomorphic bacteria pathobiology and could ultimately identify common novel antibiotic targets for treating the diseases they cause.

## INTRODUCTION

Diseases caused by intracellular bacteria that live in host cell-derived vacuoles impose immense public health, agricultural, and economic burdens worldwide. Many vacuole-adapted bacteria exhibit pleomorphic lifestyles where they transition between discrete forms that perform critical pathobiological tasks including host cell invasion and intracellular replication (1–7). However, the processes that facilitate morphotype conversion are largely undefined. Much knowledge about bacterial pleomorphism is derived from extensive research of phylogenetically diverse model systems such as the aquatic bacterium *Caulobacter crescentus*, which exists as either a sessile stalked cell or motile swarmer cell, as well as soil-dwelling *Bacillus* and *Streptomyces* spp. that transition between vegetative cells and environmentally stable spores. Notably, a common mechanism underpinning pleomorphism in each of these model organisms is cell division (8–13).

Bacterial cell division occurs either symmetrically or asymmetrically with each mode affording specific fitness advantages. In symmetrical division, also deemed binary fission, cellular components are distributed evenly across the midcell of a dividing bacterium to yield two identical daughter cells. Binary fission facilitates rapid exponential expansion of a bacterial population within a newly established microenvironment. Asymmetrical division, on the other hand, results in uneven distribution of cellular components, often genetic material, transcription factors, and proteases, to yield daughter cells with distinct phenotypic fates (14–17). By producing morphologically distinct offspring, asymmetrically dividing organisms can quickly respond to environmental stimuli to enhance their survival in disparate and changing microenvironments. Remarkably, pleomorphic bacteria have evolved to use each cell division mode either individually or in concert to balance population expansion with adaptability. As an example of the former, the *C. crescentus* stalked mother cell divides exclusively via asymmetrical division to produce swarmer cells capable of dispersion to nutrient replete environments (8, 9). In contrast, *Bacillus* spp. and the vacuolar-adapted obligate intracellular pathogen *Chlamydia trachomatis*, use both modes, undergoing symmetrical division for rapid population expansion and asymmetrical division to produce non-replicative persistent forms (10, 12, 18–21). Whether cell division is linked to morphotype conversion in vacuolar-adapted pathogens besides *Chlamydia* and, if so, which mode(s) are involved has yet to be investigated.

*Anaplasma phagocytophilum* is a pleomorphic obligate intracellular bacterium that resides in a pathogen-modified vacuole for its entire lifecycle except for a brief extracellular period when it disseminates for infection (22). *A. phagocytophilum* naturally cycles between ixodid ticks and small mammalian reservoirs (23). *A. phagocytophilum* causes granulocytic anaplasmosis in humans, horses, and companion animals and tickborne fever in ruminants (24). Human granulocytic anaplasmosis (HGA) is a globally emerging disease and currently the most reported rickettsial illness in the United States with 19,825 reported cases from 2019 to 2022 (25, 26). HGA presents as an acute febrile illness that can progress to severe disease requiring intensive care. Symptoms and complications can include leukopenia, thrombocytopenia, rhabdomyolysis, transaminitis, splenomegaly, increased susceptibility to opportunistic infections, multiorgan failure, shock, and death (26, 27). Productive *A. phagocytophilum* infection, and thus disease, wholly relies on completion of the bacterium’s biphasic developmental cycle, where it transitions between two distinct morphotypes: the infectious, non-replicative dense-cored (DC) form and the non-infectious, replicative reticulate cell (RC) form (1). The DC is approximately 0.5 µm in diameter, has an electron-dense nucleoid, and is enriched in outer membrane-localized adhesins and invasins (1, 28–30). The DC invades host cells via receptor-mediated endocytosis to reside within a pathogen-modified multivesicular body referred to as the ApV (*A. phagocytophilum* vacuole) (29, 31–34). After invasion, the DC de-condenses its nucleoid and increases in size to approximately 1.0–2.0 µm to convert to the RC form (1). The RC proceeds through successive rounds of replication to increase bacterial numbers before the resulting population transitions back to DCs for exocytic release and reinfection (1, 22). The mechanisms that control morphotype development are unknown.

In this study, we interrogated the role of cell division throughout the *A. phagocytophilum* developmental cycle. Our data suggest that development is synchronous through the RC replication phase but becomes asynchronous upon RC-to-DC differentiation and ApV exocytosis. Alternative modes of cell division appear to be utilized during specific developmental phases, where RCs replicate by binary fission and DCs are produced by an asymmetric division process reminiscent of canonical endospore formation. The actin-homolog and elongation protein, MreB, is critical for *A. phagocytophilum* cell division, specifically septation. Lastly, inhibition of cell division prevents DC formation, ApV maturation, and bacterial dissemination. Overall, *A. phagocytophilum* cell division is a key driver in RC-to-DC conversion and infection cycle progression.

## RESULTS

### Development of a reinfection assay for *A. phagocytophilum* titer quantification

**Fig 1:**
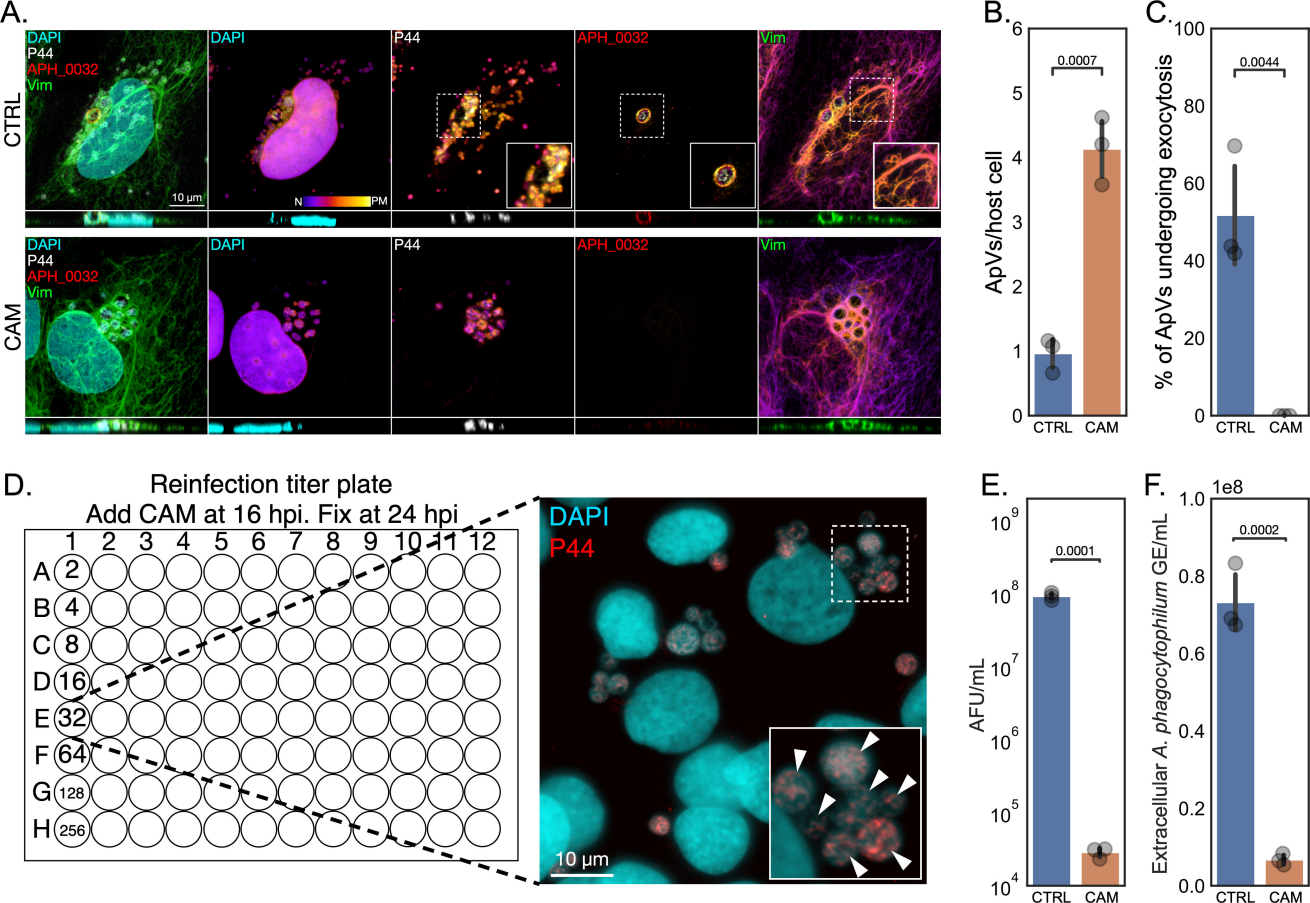
Construction of an *A. phagocytophilum* reinfection assay. (**A**) to (**C**) Inhibition of late-stage *A. phagocytophilum* protein synthesis prevents ApV exocytosis and reinfection. RF/6A cells were synchronously infected with *A. phagocytophilum*. Infected cells were treated with vehicle (CTRL) or chloramphenicol (CAM) at 16 hpi. At 26 hpi, samples were fixed, stained with DAPI (host cell nucleus and bacterial nucleoids), and immunolabeled for P44 (individual *A. phagocytophilum* organisms), APH_0032 (late-stage ApV membrane), and vimentin (Vim [encircles individual ApVs]) and visualized by laser-scanning confocal microscopy. (**A**) Representative Z-projection confocal micrographs. The Z-projections represent successive Z-plane images (17 for CTRL and 28 for CAM). For the individual channel panels (DAPI, P44, APH_0032, and Vim), a color code, which is indicated at the bottom of the DAPI panel, denotes the three-dimensional depth of each channel starting from the bottom (nucleus [N]) to top (plasma membrane [PM]) of the host cell. The regions that are denoted by hatched lined boxes are magnified in the insets demarcated by solid lined boxes. An orthogonal view of the Z-stack is provided below each Z-projection. Scale bar, 10 µm. Translucent squares have been placed behind the colored text in the micrographs to increase visibility of the text. (**B**) The mean (± SD) number of ApVs per host cell was determined by examining at least 100 ApVs per condition. (**C**) The mean (± SD) percentage of exocytic ApVs per host cell was calculated by dividing emptied ApVs by the total number of ApVs. (**D**) to (**F**) Reinfection assay. (**D**) Schematic of the reinfection assay and representative output immunofluorescence micrograph. Host cell-free media from synchronously infected CTRL or CAM treated RF/6A cells was collected at 26 hpi, a time point at which the extracellular milieu would normally contain exocytosed *A. phagocytophilum* DCs, and added as twofold serial dilutions in a volume of 100 µL to confluent naïve RF/6A cells in a microtiter plate. The reinfection microtiter plates were treated with CAM at 16 hpi to prevent ApV exocytosis followed by fixation at 24 hpi, DAPI staining, P44 immunolabeling, and immunofluorescence microscopy imaging. Individual ApVs, visualized and identified by colocalization of DAPI stained nucleoids with P44 immunosignal, were counted using Fiji. In the representative micrograph, arrowheads within the enlarged inset denote individual ApVs that would be counted as a single ApV-forming unit (AFU). Scale bar,10 µm. (**E**) The mean AFU/mL was determined via back calculation based on dilution and microtiter well surface area. (**F**) The mean (± SD) *A. phagocytophilum* GE/mL was determined by qPCR from DNA isolated from host cell-free media from the initial infection. Data are means ± SD of three experimental replicates per condition. Independent *t*-tests were performed for statistical analysis.

*A. phagocytophilum* morphotype transition and growth occur in three broadly defined stages: DC-to-RC differentiation, RC replication, and RC-to-DC conversion (1, 28, 35, 36). To properly assess the contributions of cell division to *A. phagocytophilum* development, we needed an approach for accurately monitoring the temporal dynamics of morphotype transitioning. We constructed a reinfection assay leveraging two aspects of the infection cycle. First, ApVs do not undergo homotypic fusion (37). Hence, each ApV derives from a single bacterium and can serve as a proxy for quantifying inocula. Second, following RC-to-DC conversion ApVs undergo exocytosis to release DCs (22), which reduces the number of ApVs per host cell and thereby obfuscates infectious titer quantification. As DC morphotype development corresponds temporally to ApV exocytosis (22, 36, 38), we rationalized that inhibiting late-stage bacterial protein synthesis would prevent ApV exocytosis. Accordingly, we synchronously infected RF/6A choroidal endothelial cells and added chloramphenicol at 16 h post-infection (hpi). RF/6A cells were used because they are well-established for studying *A. phagocytophilum*-host cell interactions, and their flat morphology allows for excellent ApV visualization using microscopy (22, 38–40). At 26 hpi, a time point coincident with *A. phagocytophilum* release (22), samples were fixed, stained with 4′,6-diamidino-2-phenylindole (DAPI) to label host and *A. phagocytophilum* DNA, and immunolabeled for P44, APH_0032, and vimentin. P44 is an *A. phagocytophilum* outer membrane protein (OMP) expressed throughout infection (28). APH_0032 is a late-stage bacterial effector that is expressed beginning at ~18 h and localizes to the ApV membrane (35). Vimentin is a host cell intermediate filament protein that wraps ApVs and is useful for vacuole visualization (39). Confocal Z-stack images were taken and the resulting Z-projections color coded for depth visualization (22). As expected, control cells exhibited a subset of APH_0032-positive vacuoles that were located at the plasma membrane and devoid of *A. phagocytophilum* organisms (Fig. 1A). These characteristics are consistent with exocytic release of DCs from ApVs that had trafficked to the plasma membrane (22). Indeed, adjacent to the emptied ApVs were numerous smaller ApVs each of which harbored a single bacterium, indicating that exocytosed DCs had initiated reinfection. In chloramphenicol-treated cells, all the ApVs were APH_0032-negative, perinuclear, and filled with bacteria. Additionally, there were no signs of reinfection. Chloramphenicol promoted ApV retention to increase the ApV number per host cell by fourfold (Fig. 1B). Moreover, whereas 51% of ApVs in controls had undergone exocytosis, no exocytic ApVs were present in chloramphenicol-treated cells (Fig. 1C). These results confirm that inhibiting bacterial protein synthesis prevents ApV exocytosis and provides a platform for quantifying infectious titer.

Armed with this information, we constructed the reinfection assay (Fig. 1D). Media from RF/6A cultures that had been synchronously infected and treated with chloramphenicol or vehicle beginning at 16 hpi were collected at 26 hpi and divided into two aliquots. The first was used to determine DC progeny number in the extracellular milieu by adding twofold serial dilutions to naïve RF/6A cells in a microtiter plate. At 16 h post-reinfection, all samples in the plate were treated with chloramphenicol to enable DC titer assessment. At 24 h post-reinfection, the samples were fixed, stained with DAPI, immunolabeled for P44, and examined by immunofluorescence microscopy. Chloramphenicol treatment led to a more than three-log decrease in infectious progeny upon reinfection (Fig. 1E). The second aliquot was analyzed by qPCR, which showed that the number of *A. phagocytophilum* genome equivalents (GE) and hence released bacteria in media of chloramphenicol treated cells were reduced several-fold (Fig. 1F). These results provide proof-of-concept that this assay can effectively monitor *A. phagocytophilum* DC development, release, and titer.

### The *A. phagocytophilum* developmental cycle consists of synchronous and asynchronous phases

**Fig 2:**
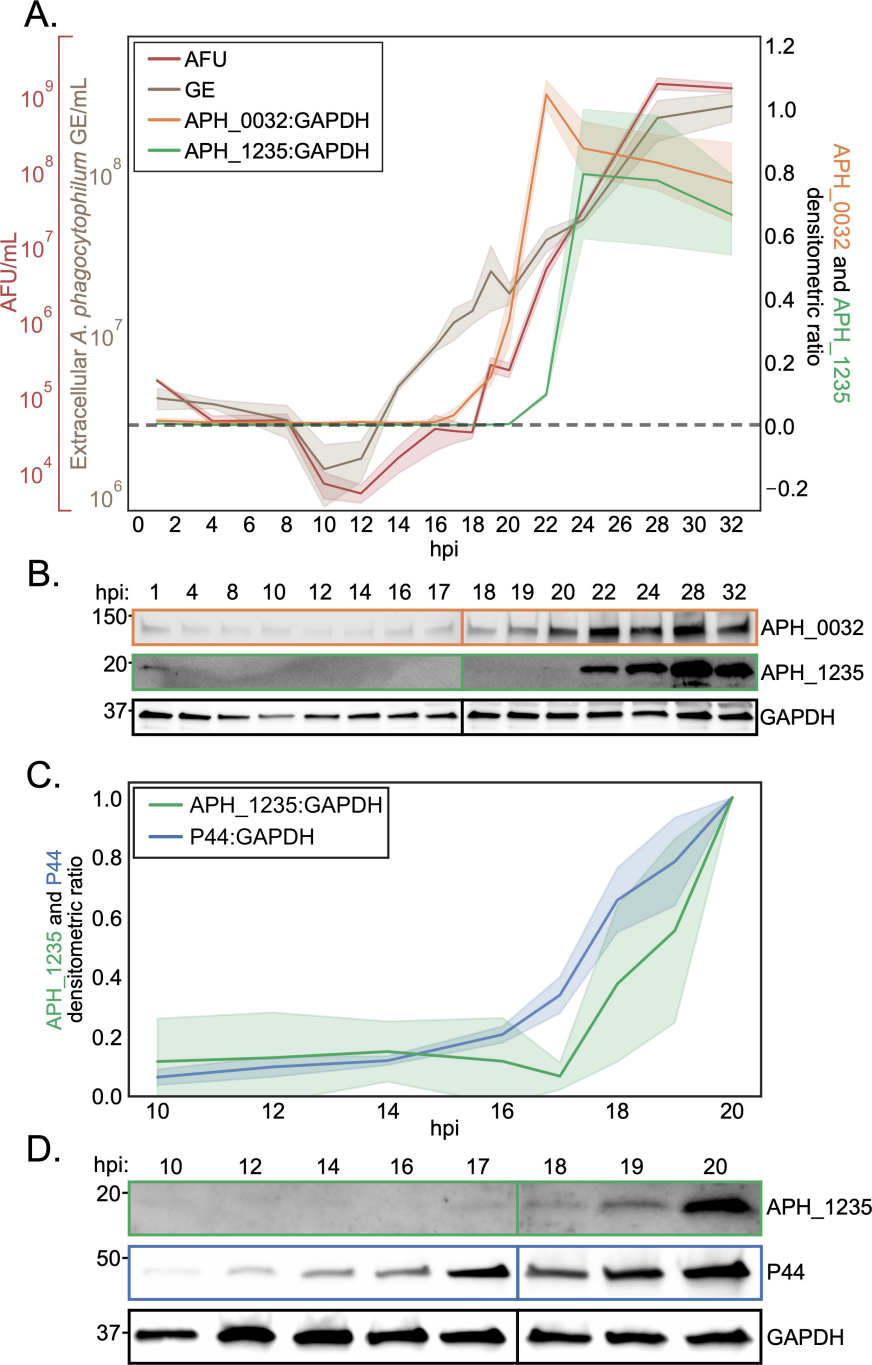
Temporal dynamics of *A. phagocytophilum* late-stage development. RF/6A cells were synchronously infected with *A. phagocytophilum*. At the indicated hpi, DCs that had been exocytosed into the media were collected and AFU/mL and *A. phagocytophilum* GE/mL were enumerated by reinfection assay or qPCR using primers targeting *A. phagocytophilum dnaK*, respectively. Protein isolated from infected cultures (media and cell monolayers) at the indicated hpi were subjected to western blot analysis using antisera and antibody against APH_0032, APH_1235, and GAPDH. (**A**) The temporal dynamics of AFU/mL and *A. phagocytophilum* GE from the extracellular milieu along with the APH_0032:GAPDH and P44:GAPDH densitometric signal ratios from infected cells are presented. The dotted line represents AFU and GE baselines prior to reinfection and *A. phagocytophilum* release, respectively. (**B**) Representative western blots of APH_1235, APH_0032, and GAPDH from which densitometric ratio values presented in (**A**) were calculated. (**C**) Lysates from (**A**) at the indicated time points were reassessed by western blot analysis to determine the APH_1235:GAPDH and P44:GAPDH densitometric ratios. (**D**) Representative western blots of APH_1235, P44, and GAPDH from which densitometric ratio values presented in (**C**) were calculated. In (**A**) and (**C**), western blot colors correspond to line plot colors, solid lines represent the mean of experimental replicates, and clouds represent standard deviation. Data are means ± SD of three experimental replicates per sample.

Prior analysis in which *A. phagocytophilum* infected cells were examined at 4 to 8 h intervals suggested that RC-to-DC conversion begins at 24 hpi (36). However, our recent study showed that ApV exocytosis initiates by 22 hpi, suggesting that DC differentiation begins even earlier (22). To refine our understanding of the temporal dynamics of *A. phagocytophilum* development, we coupled the reinfection assay and qPCR with immunoblot analyses as well as assessed more frequent time points between 1 and 32 hpi. In particular, we focused on single-h intervals between 16 hpi, when all *A. phagocytophilum* organisms are RCs (36), and 20 hpi, a time point that we speculated precedes ApV exocytosis. *A. phagocytophilum* GE in the extracellular milieu increased by one log above baseline at 18 h, and an increase in ApV forming units (AFU) was first detected between 18 and 19 hpi (Fig. 2A). Remarkably, the number of infectious DC progeny released into the media increased by more than four logs between 18 and 28 hpi. Western blots were probed for APH_0032 as a late infection marker and APH_1235, an *A. phagocytophilum* OMP that is exclusive to DCs (28, 35, 36). Consistent with prior studies (35, 36), APH_0032 protein levels began to increase around 17 hpi and remained elevated for the rest of the time course (Fig. 2A and B). APH_1235 was first observed at 20 hpi, 4 h earlier than previously reported (28). We posited that the abundant APH_1235 immunosignal at 22 through 32 hpi could have impeded our ability to observe low-level APH_1235 expression before 20 h due to the fact that AFUs were released into the media beginning at 18 hpi. Indeed, western blot analysis performed on 10 through 20 hpi samples detected APH_1235 at 18 hpi (Fig. 2C and D).

**Fig 3:**
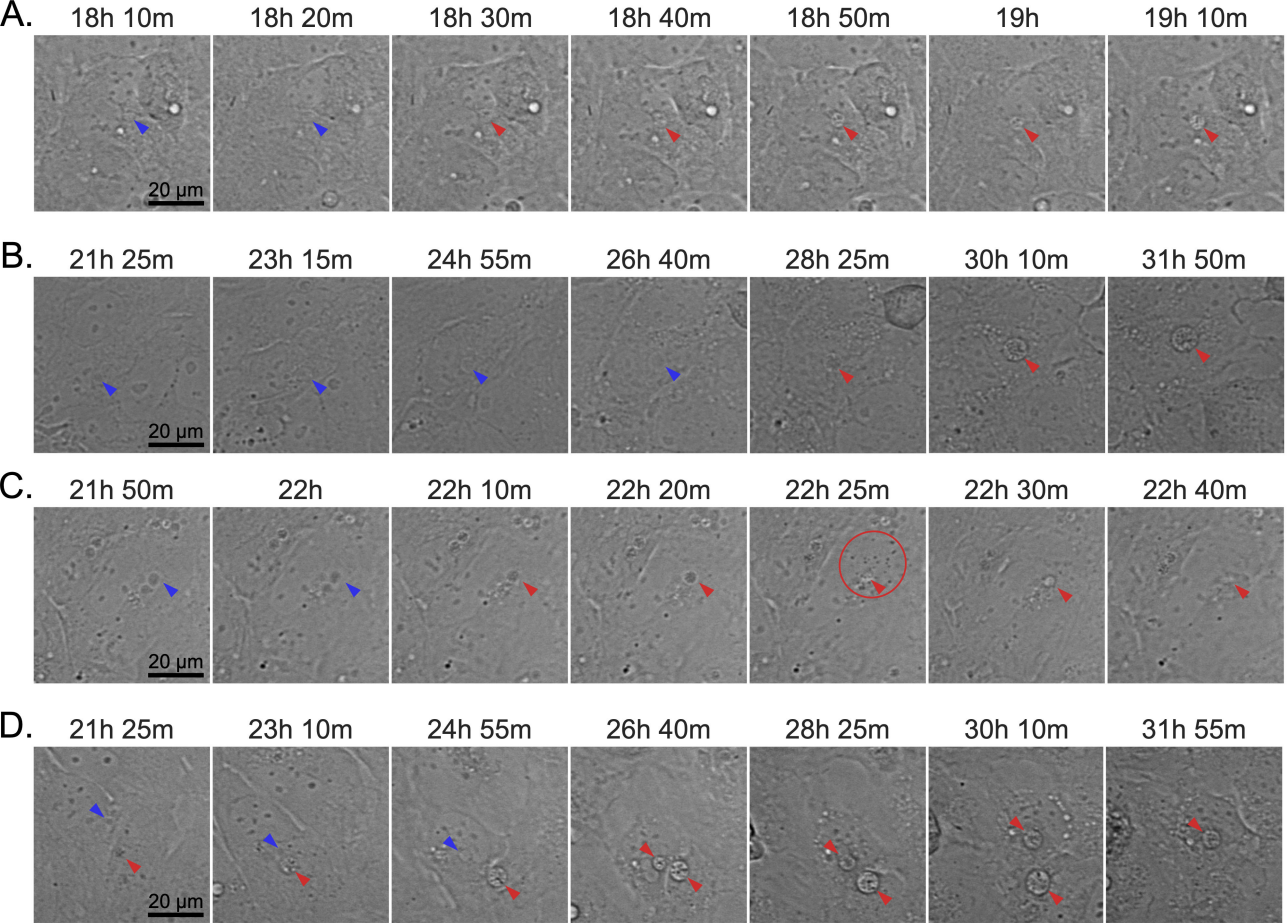
Live-cell microscopy of individual ApV development and exocytosis. Live-cell microscopy was performed on synchronously infected RF/6A cells from 16 to 35 hpi. Panels (**A**) to (**D**) correspond with Movies S1 to S4, respectively. Representative snapshot micrographs display RC-to-DC conversion and bacterial release from individual ApVs. Blue and red arrowheads denote individual ApVs containing RCs or DCs, respectively. The red circle in panel (**C**) in the 22 h 25 min frame surrounds released DCs. Scale bars, 20 µm.

**Fig 4:**
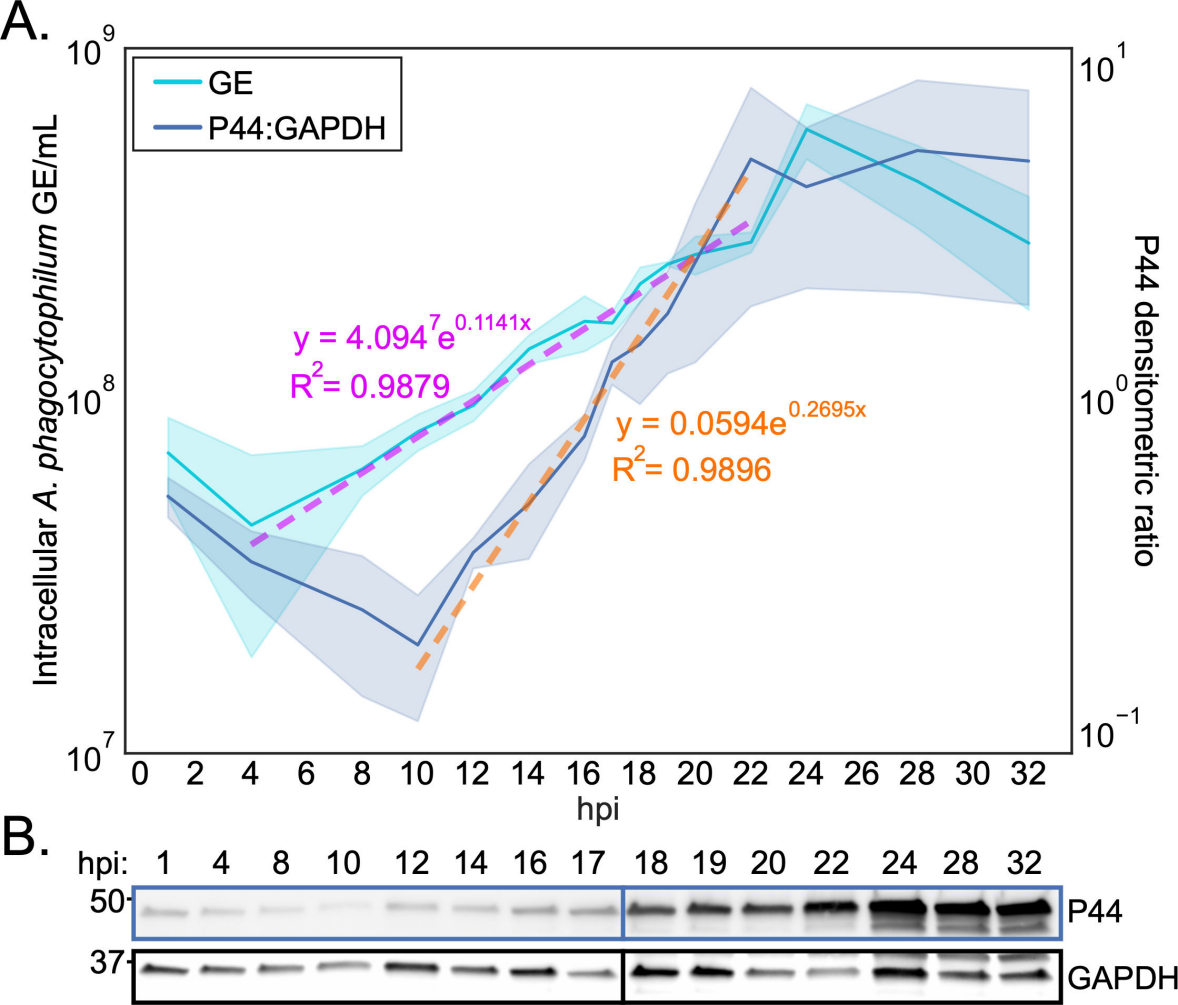
*A. phagocytophilum* replication dynamics. Total DNA and protein recovered from monolayers of synchronously infected RF/6A cells at the indicated hpi were subjected to qPCR using primers targeting *A. phagocytophilum dnaK* or western blot analysis using antiserum and antibody against P44 and GAPDH, respectively. The temporal dynamics of *A. phagocytophilum* GE and P44:GAPDH densitometric signal ratio are graphed. Solid lines represent the mean of experimental replicates, while clouds represent standard deviation. The dotted magenta and orange lines represent linear regression fits from 4 to 22 (GE) and 10 to 22 (P44:GAPDH) hpi, respectively. (**B**) Representative western blots of P44 and GAPDH from which the P44:GAPDH densitometric ratio values presented in (**A**) were calculated. Data are means ± SD of three experimental replicates per sample.

We next interrogated DC developmental and ApV exocytosis dynamics at the level of individual ApVs using live-cell time-lapse microscopy. Phase images were taken at 5 min intervals from 16–35 hpi. We found RC-to-DC conversion to be highly asynchronous. For instance, some RCs developed into DCs as early as between 18 and 19 hpi (Fig. 3A; Movie S1), while others did not transition until after 28 hpi (Fig. 3B; Movie S2). ApV maturation and bacterial release were also asynchronous with an ApV subpopulation undergoing exocytosis in as little as 15 min post-DC conversion (Fig. 3C; Movie S3), while other ApVs remained inside host cells for more than 10 h even though they contained DCs (Fig. 3D; Movie S4). Lastly, we surveyed *A. phagocytophilum* replication. P44 expression began to increase at 12 hpi and plateaued at 22 hpi (Fig. 4). *A. phagocytophilum* GE increased after 4 hpi and rose until 24 hpi after which they sharply decreased likely due to mass exocytosis of bacteria. The P44 and *A. phagocytophilum* GE increases were both exponential, suggesting that RCs replicate by binary fission. Collectively, these data demonstrate that the *A. phagocytophilum* infection cycle begins synchronously with a monophasic RC replication phase that spans from 4 to 18 hpi after which DC conversion and ApV exocytosis each proceed asynchronously.

### Differential modes of *A. phagocytophilum* cell division are morphotype specific

**Fig 5:**
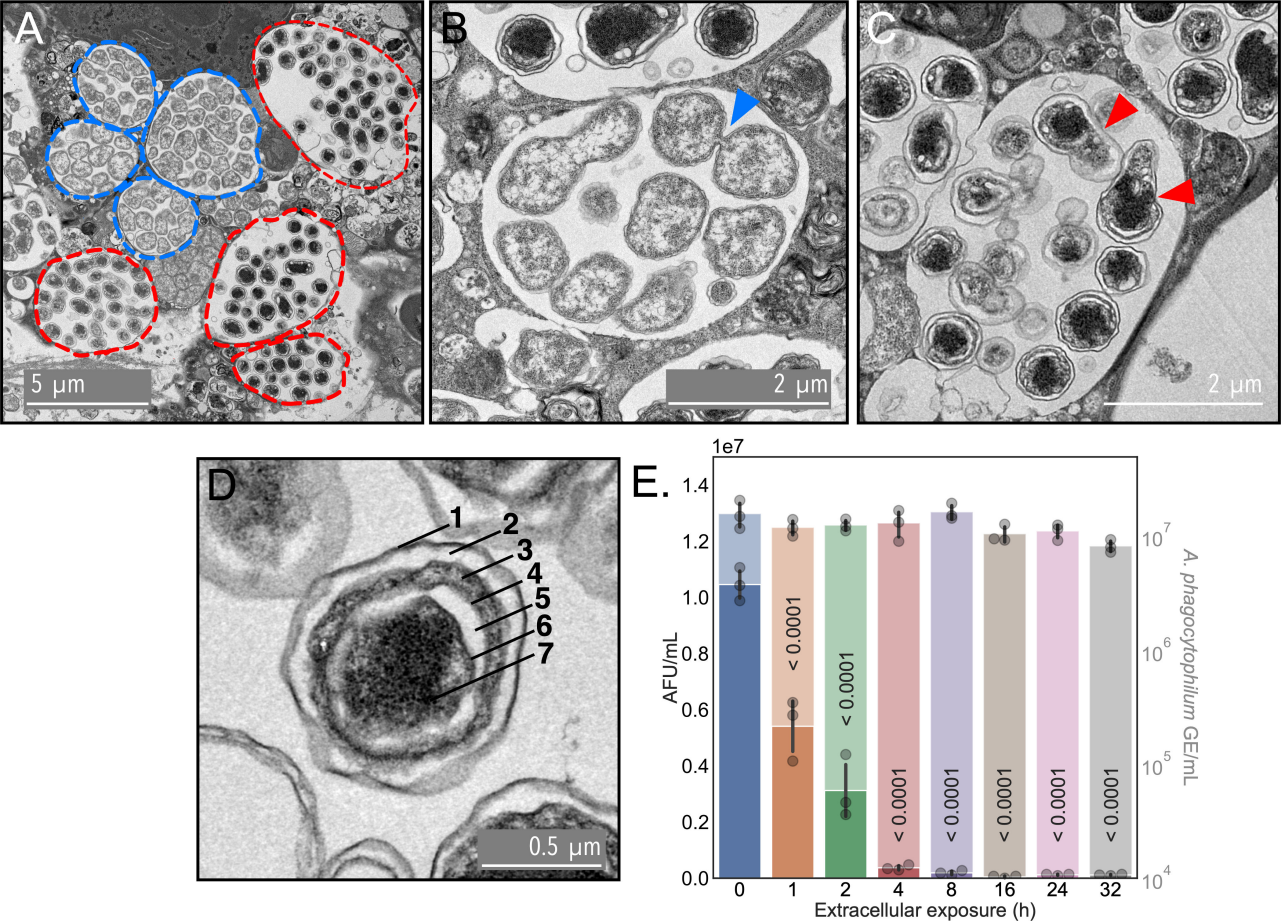
*A. phagocytophilum* RCs and DCs undergo distinct forms of cell division. (**A**) to (**D**) RF/6A cells synchronously infected with *A. phagocytophilum* were visualized by transmission electron microscopy at 24 hpi. (**A**) The ApVs encircled by hatched blue and red lines contain RCs or DCs, respectively. The blue arrowhead in panel (**B**) indicates the septum between two RC bacteria dividing by binary fission. Red arrowheads in (**C**) denote two bacteria undergoing RC-to-DC conversion via an asymmetric division process reminiscent of sporulation. Note that both bacteria contain electron-dense regions consistent with the canonical DC morphotype but also have electron lucent RC-like tails that superficially resemble mother cell lysis during sporulation. (**D**) A single DC organism demonstrating multi-layered subcellular structures that resemble the layers of a canonical endospore: exosporium (1), interspace (2), coat (3), outer membrane (4), cortex (5), inner membrane (6), and core (7). Scale bars in (**A**), (**B**), (**C**), and (**D**) are 5, 2, 2, and 0.5 µm, respectively. (**E**) Extracellular *A. phagocytophilum* DCs rapidly lose infectivity. DCs that had been naturally released from host cells were incubated extracellularly for the indicated periods before infection capabilities were assessed using the reinfection assay. AFU/mL and *A. phagocytophilum* GE/mL, denoted by opaque and transparent bars, respectively, were determined. Data are means ± SD of three experimental replicates per time point. A one-way analysis of variance (ANOVA), followed by a Tukey’s multiple comparisons analysis, was performed for statistical analysis. The reinfection ANOVA had degrees of freedom of 7, 16; the *F*-statistic equaled 119.8; and the *P*-value was less than 0.0001. For the GE ANOVA, the degrees of freedom were 7, 16; the *F*-statistic equaled 3.019; and the *P*-value equaled 0.031852. The adjusted *P*-values for the reinfection assay multiple comparisons analysis are presented on the graph.

Next, we examined cell division during morphotype growth and development by visualizing RC and DC ultrastructure in infected host cells via transmission electron microscopy (TEM). As RC-to-DC conversion is highly asynchronous but does occur at 24 hpi, we focused on this time point. The vast majority of ApVs contained only RCs or DCs (Fig. 5A). RCs were observed dividing by what appeared to be binary fission (Fig. 5B). Intriguingly, DC division appeared asymmetric and resembled mother cell lysis during endospore formation (Fig. 5C) (41, 42). Individual DC ultrastructure superficially resembled the seven layers of a canonical *Bacillus* endospore: exosporium, interspace, coat, outer membrane, cortex, inner membrane, and core (43) (Fig. 5D). To determine if DCs, like endospores, are capable of long-term extracellular survival, DCs that had been naturally exocytosed were recovered and placed in tissue culture media and incubated at 37°C in 5% atmospheric CO_2_. At 0, 1, 2, 4, 8 16, 24, and 32 h, aliquots were assayed for their reinfection capabilities and their *A. phagocytophilum* GE quantified. Although bacterial GEs were consistent across time points, infectivity decreased by 48.3% after only 1 h of extracellular exposure and continued to drop until it was below the level of detection by 8 h (Fig. 5E).

**Fig 6:**
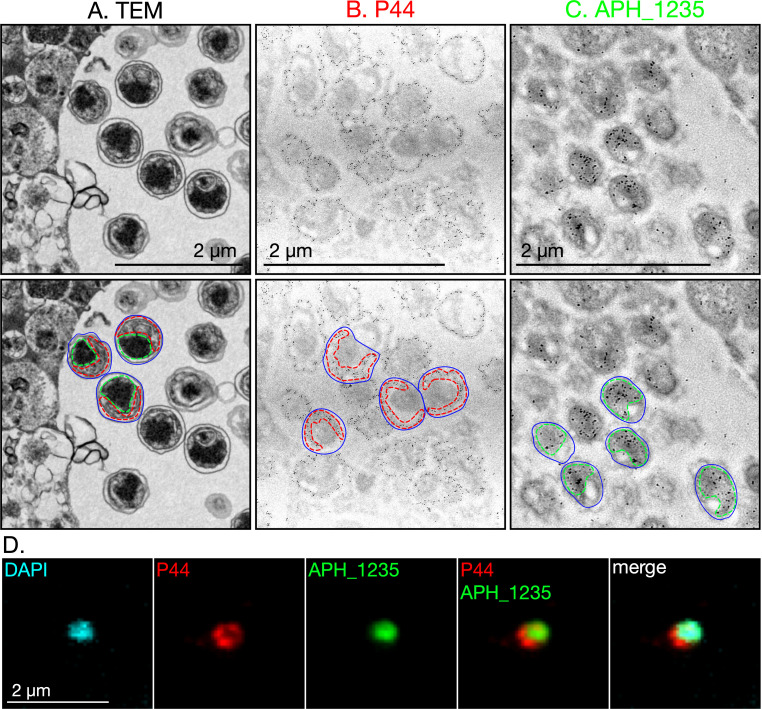
The *A. phagocytophilum* DC morphotype is polarized. (**A**) *A. phagocytophilum* infected RF/6A cells were fixed and examined by TEM. The upper panel is a transmission electron micrograph showing an ApV full of DCs at 24 hpi. The lower panel presents the same image with polarity indicated on three representative DCs. The outer edge of each bacterium is outlined by a blue line. The electron-lucent and electron-dense regions are demarcated by red and green dotted lines, respectively. (**B**) and (**C**) Assessment of P44 and APH_1235 polar distribution on DCs by immunoelectron microscopy. *A. phagocytophilum* infected HL-60 cells were fixed and screened with anti-P44 (**B**) or anti-APH_1235 (**C**) followed by goat anti-mouse IgG conjugated to 6 nm gold particles and examined by electron microscopy. Duplicate images of the upper panel micrographs are overlaid in the lower panel with the outer edges of representative DCs outlined in blue. Red and green dotted lines in (**B**) and (**C**) denote P44 and APH_1235 immunogold labeling patterns, respectively. (**D**) Assessment of P44 and APH_1235 polar distribution on DCs by immunofluorescence microscopy. Infected RF/6A cells were fixed, stained with DAPI, and immunolabeled for P44 and APH_1235. Shown is a representative immunofluorescence micrograph of a single DC organism. Scale bars, 2 µm. Data are representative of more than three independent experiments.

As cell division can lead to the polarization of proteins on and within cells (9, 44), we assessed if DCs are polarized. Per TEM visualization, individual DCs exhibited opposing electron-dense and electron-lucent regions (Fig. 6A). Immunogold labeling revealed that the electron-lucent and electron-dense regions were exclusively P44- or APH_1235-positive, respectively (Fig. 6B and C). Immunofluorescence micrographs showed similar stratification of P44 and APH_1235 immunosignal, where APH_1235 colocalized with the DAPI-stained nucleoid and P44 localized to the opposite pole (Fig. 6D). Overall, these results support that, while RC appear to undergo binary fission, RC-to-DC conversion likely occurs via asymmetric division similar to that of sporulation. However, unlike canonical endospores, DCs are not environmentally stable.

### MreB is critical for *A. phagocytophilum* septation

**Fig 7:**
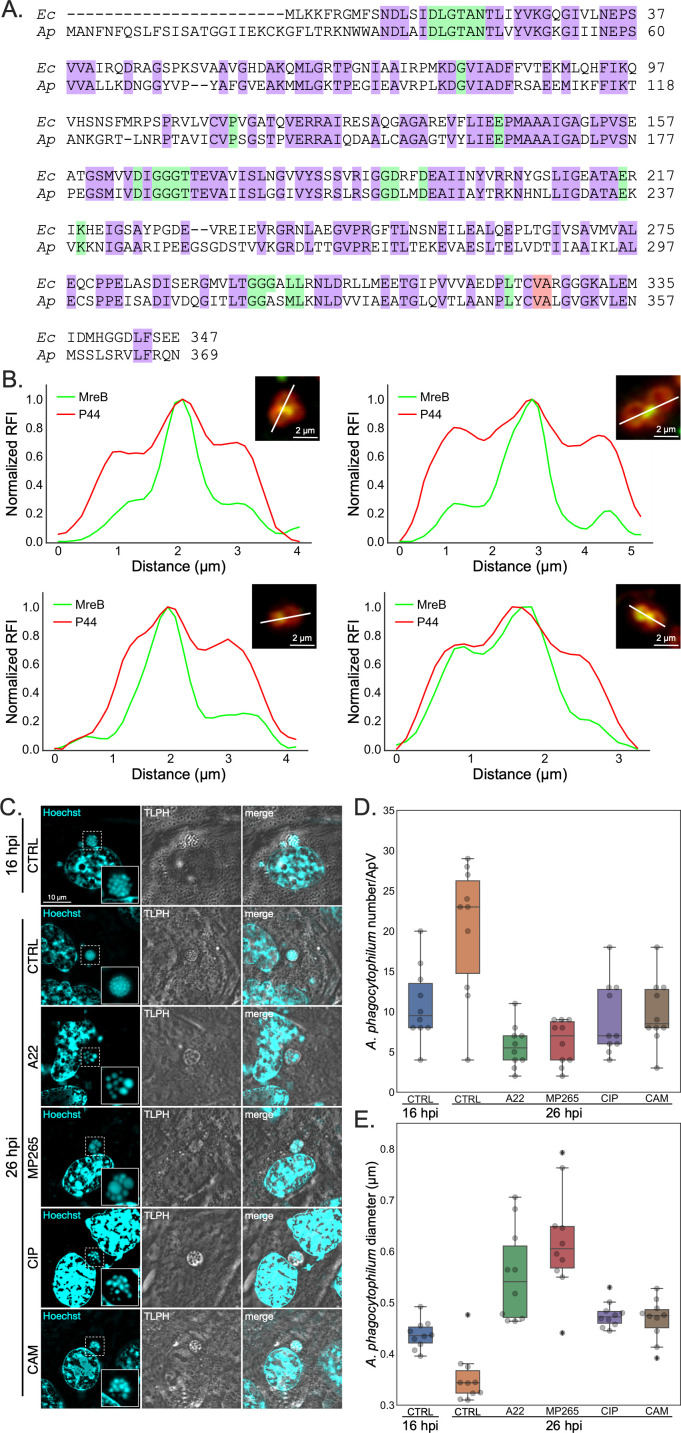
MreB is key for *A. phagocytophilum* septation. (**A**) Amino acid alignment of MreB from *E. coli* str. K12 (Ec [Accession: NP_417717, GeneID: 948588]) and *A. phagocytophilum* str. NCH-1 (Ap [Accession: KJV63182; GeneID: EPHNCH_1088]). Alignment was performed using the Clustal Omega Multiple Sequence Alignment tool by EMBL-EBI. Identical residues are highlighted purple. Green highlighted residues correspond to known MreB active sites based on *T. maritima* and/or *S. cerevisiae* MreB crystal structures. Red highlighting denotes the A22 target site. (**B**) MreB immunosignal localizes to the septum of dividing *A. phagocytophilum* organisms. RCs isolated from synchronously infected RF/6A cells at 16 hpi were fixed, immunolabeled for MreB and P44, and assessed on a single cell basis for MreB subcellular localization. Representative micrographs and associated lateral line plots of four dividing bacteria across the division septum are shown. Fluorescent intensities of the MreB and P44 channels were normalized from 0 to 1 and are denoted as normalized relative fluorescent intensity (RFI). (**C**) to (**E**) MreB activity and *A. phagocytophilum* DNA and protein synthesis are critical for division. Synchronously infected RF/6A cells were treated at 16 hpi with vehicle (CTRL), A22, MP265, ciprofloxacin (CIP), or chloramphenicol (CAM). At 16 or 26 hpi, cells were stained with Hoechst 33342 and imaged. (**C**) Representative live-cell fluorescent (Hoechst), transmitted light phase (TLPH), and merged images are presented. The regions containing individual ApVs that are denoted by hatched lined boxes are magnified in the insets that are demarcated by solid lined boxes. (**D**) and (**E**) 10 ApVs per condition were assessed to determine the number and size of bacteria. For each boxplot value, the horizontal gray line represents the median while the boxes represent the 25th to 75th percentiles.

We next sought to interrogate the functional relevance of cell division for *A. phagocytophilum* pathobiology. Genome analyses predict that the bacterium lacks most canonical divisome machineries (45, 46). Those that is does encode are likely essential. Moreover, *A. phagocytophilum* is genetically intractable (47). Therefore, instead of employing genetic approaches, we targeted the actin homolog, MreB. This protein has long been associated with elongation in rod-shaped bacteria but has recently been substantiated to aid in cell division in a variety of bacteria (48–55). *A. phagocytophilum* MreB (MreB*^Ap^*) is 52.9% identical to *Escherichia coli* MreB (MreB*^Ec^*) (Fig. 7A). Moreover, 92.9% amino acid identity is maintained within the MreB*^Ap^* known active sites when compared to *Thermotoga maritima* and/or *Saccharomyces cerevisiae*, including the target site for the antibiotic A22 (49, 56, 57). As a first step in verifying MreB involvement in *A. phagocytophilum* cell division, we visualized its subcellular localization by immunolabeling host cell-free RCs with a commercially available MreB*^Ec^* antibody in conjunction with anti-P44. Plotting the normalized relative fluorescence intensity across multiple dividing RCs revealed increased abundance of MreB at the septal plane when compared to the periphery (Fig. 7B). This observation suggests the involvement of MreB in *A. phagocytophilum* cell division.

Next, *A. phagocytophilum* infected RF/6A cells were treated with A22 or its structural analog, MP265 (58, 59), beginning at 16 hpi and imaged immediately or at 26 hpi. A22 and MP265 concentrations were at or below those used against other obligate intracellular bacteria (51, 52, 60). *A. phagocytophilum* cell size and number were assessed during live infections using the membrane-permeable DNA dye, Hoechst 33342, as a volumetric stain. A22 and MP265 reduced the bacterial numbers per ApV and induced aberrantly large cells, suggesting that the individual bacteria were still capable of increasing biomass but incapable of septation (Fig. 7C and D). To see if the latter phenotype was specific to A22 and MP265 treatment, we utilized ciprofloxacin, which inhibits DNA gyrase and topoisomerase to prevent bacterial DNA replication (61), and chloramphenicol. Although both antibiotics prevented bacterial replication (Fig. 7C and D), the sizes of individual *A. phagocytophilum* bacteria were unchanged relative to the 16 hpi control (Fig. 7C and E). These results support that MreB is an *A. phagocytophilum* cell division protein that A22 and MP265 inhibit to prevent septation.

### *A. phagocytophilum* cell division is required for DC formation

**Fig 8:**
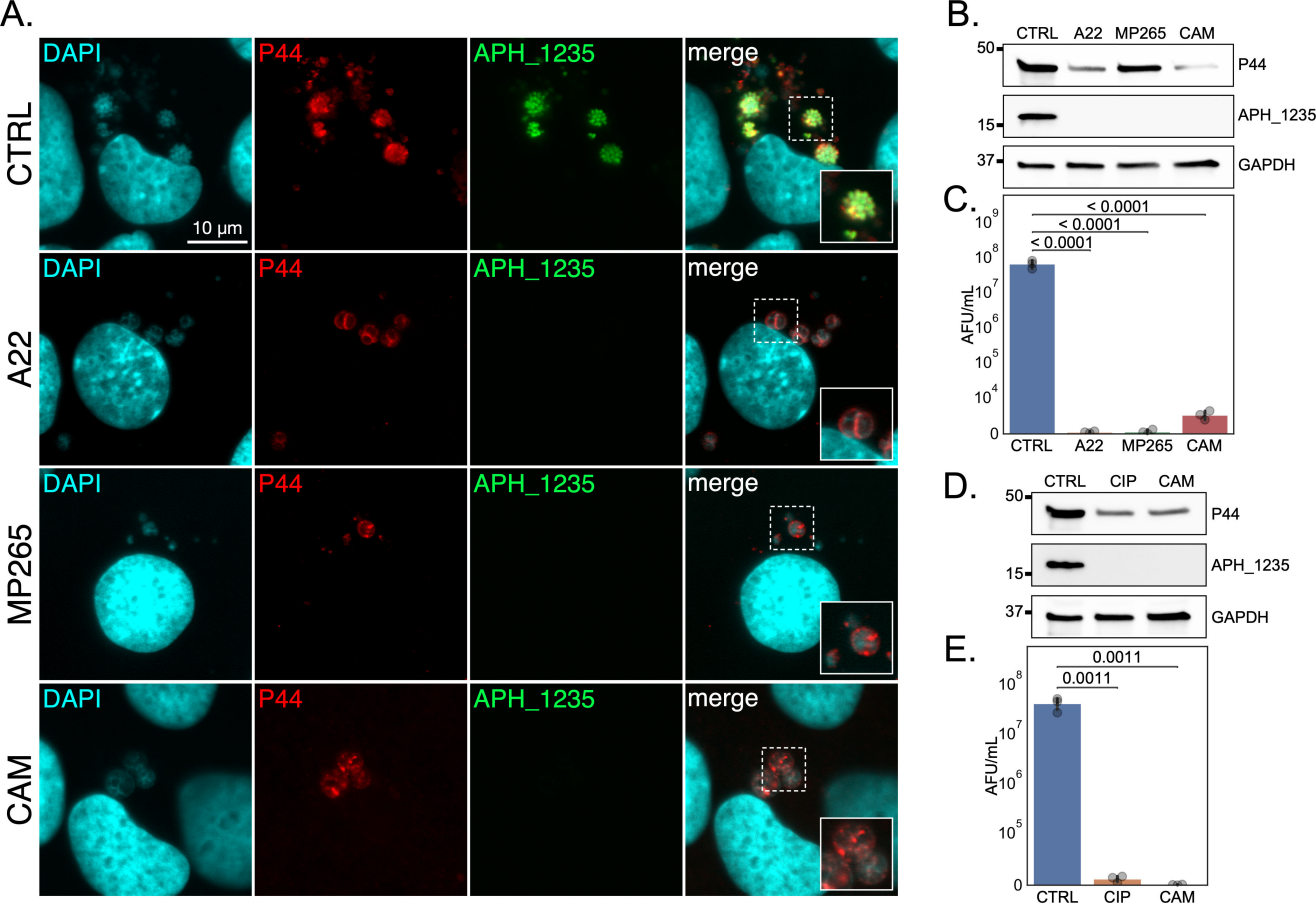
Cell division inhibition prevents DC formation. RF/6A cells were synchronously infected with *A. phagocytophilum*. (**A**) to (**C**) Infected cells were treated with vehicle (CTRL), A22, MP265, or chloramphenicol (CAM) at 16 hpi. At 26 hpi, the cells were (**A**) fixed, stained with DAPI, immunolabeled for P44 and APH_1235, and examined by immunofluorescence microscopy. The regions that are denoted by hatched lined boxes are magnified in the insets demarcated by solid lined boxes. Alternatively, (**B**) cells were lysed followed by western blot analyses assessing P44, APH_1235, and GAPDH protein levels or (**C**) host cell-free media was collected and DCs released into the extracellular milieu were measured as AFU/mL using the reinfection assay. (**D**) and (**E**) Synchronously infected cells treated with CTRL, ciprofloxacin (CIP), or CAM at 16 hpi were either (**D**) lysed and western blot analyses performed or (**E**) media was collected and assessed for infectious progeny release by measuring AFU/mL via the reinfection assay at 26 hpi. The *y*-axes in (**C**) and (**E**) are in symlog format (0–10^4,5^ linear scale, >10^4,5^ log scale, respectively). Data are means ± SD of three experimental replicates per condition. A one-way analysis of variance (ANOVA), followed by a Tukey’s multiple comparisons analysis was performed for statistical analysis on (**C**) and (**E**). For (**C**), the ANOVA had degrees of freedom of 3, 8; the *F*-statistic equaled 44.85; and the *P*-value was less than 0.0001. For (**E**), the ANOVA had degrees of freedom of 2, 6; the *F*-statistic equaled 31.85; and the *P*-value equaled 0.0006. The adjusted *P*-values for (**C**) and (**E**) multiple comparisons analyses are presented on the graph.

**Fig 9:**
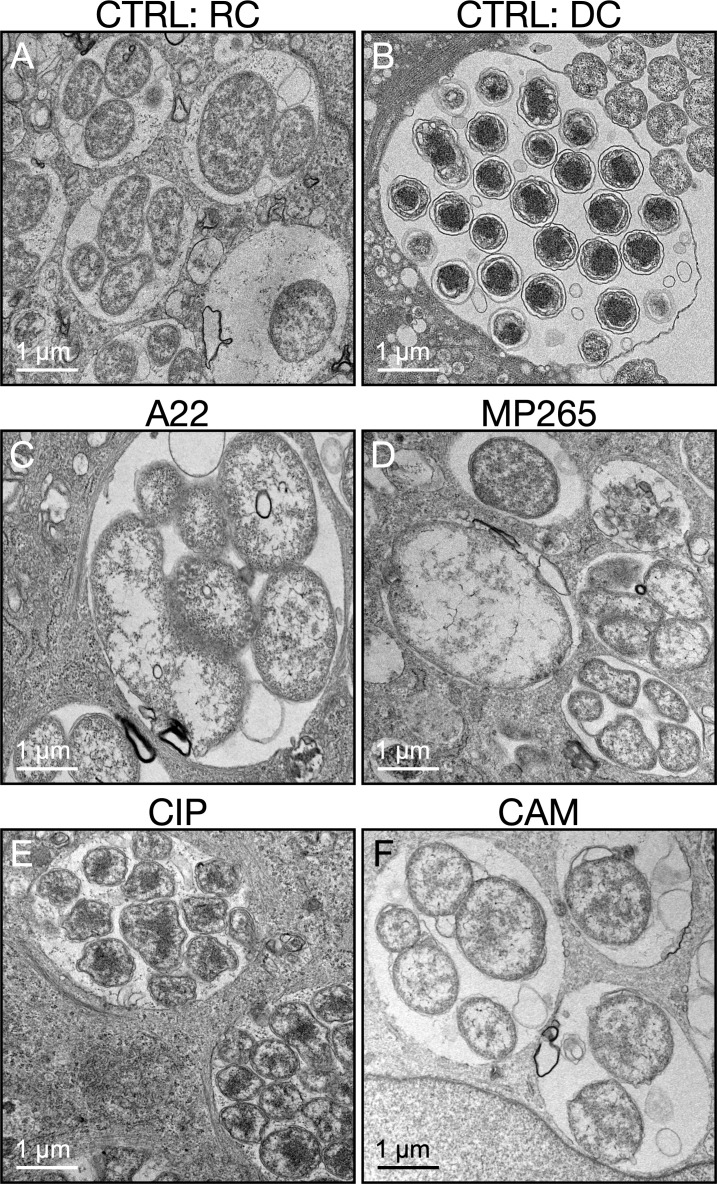
Cell division inhibition induces aberrant *A. phagocytophilum* morphology. RF/6A cells that had been synchronously infected with *A. phagocytophilum* were either fixed at 16 hpi or treated with vehicle (CTRL), A22, MP265, ciprofloxacin (CIP), or chloramphenicol (CAM) and then fixed at 26 hpi. All samples were analyzed by transmission electron microscopy. (**A**) Cells fixed at 16 hpi have ApVs that contain RCs. (**B**) ApV of a CTRL cell containing *A. phagocytophilum* bacteria exhibiting typical DC morphology. (**C**) to (**F**) ApVs of cells treated with A22 (**C**), MP265 (**D**), CIP (**E**), and CAM (**F**). Scale bars, 1 µm. Data are representative of three independent experiments.

To ascertain if septation is required for *A. phagocytophilum* DC development, synchronously infected RF/6A cells were treated with A22 or MP265 starting at 16 hpi. Chloramphenicol was included as a positive control for inhibiting RC-to-DC conversion. Similar to chloramphenicol, A22 and MP265 prevented APH_1235 expression and led to a five-log decrease in released AFUs (Fig. 8A through C). Consistent with MP265 being less cytotoxic than A22 (58, 59), P44 levels were comparable between MP265- and vehicle-treated infected cells, both of which were much higher than in A22-treated samples (Fig. 8B). Ciprofloxacin also inhibited DC formation as APH_1235 expression was undetectable and there was a four-log decrease in infectious progeny production (Fig. 8D and E). As a complementary approach, the experiment was repeated and the impact of each antibiotic on bacterial morphology was visualized by TEM. *A. phagocytophilum* cells in control conditions exhibited typical morphologies in which RCs were ~1–2 µm in diameter, electron lucent, and had a thin periplasmic space while DCs were ~0.5 µm across with electron dense cores and multiple substructural layers (Fig. 9A and B). Treatment with A22 or MP265 yielded *A. phagocytophilum* reminiscent of RCs except that they were much larger at ~4 µm in diameter (Fig. 9C and D). Ciprofloxoacin treatment resulted in bacteria that exhibited the RC-like characteristics of being ~1–2 µm across and having a thin periplasmic space but contained more electron dense nucleoids (Fig. 9E). This nucleoid condensation is likely due to the torsional strain placed on the replicating chromosome by inhibiting DNA gyrase and/or topoisomerase, as has been reported for *E. coli* and *Staphylococcus aureus* (62, 63). Chloramphenicol treatment produced *A. phagocytophilum* organisms that were slightly larger (~2 µm), many of which were halted in mid-cell division as indicated by the connected outer membrane between dividing bacteria (Fig. 9F). DCs were not observed for any antibiotic treatment. Taken together, these results establish that bacterial cell division is required for *A. phagocytophilum* RCs to initiate transition to the DC morphotype.

### *A. phagocytophilum* cell division is required for ApV maturation and DC release

**Fig 10:**
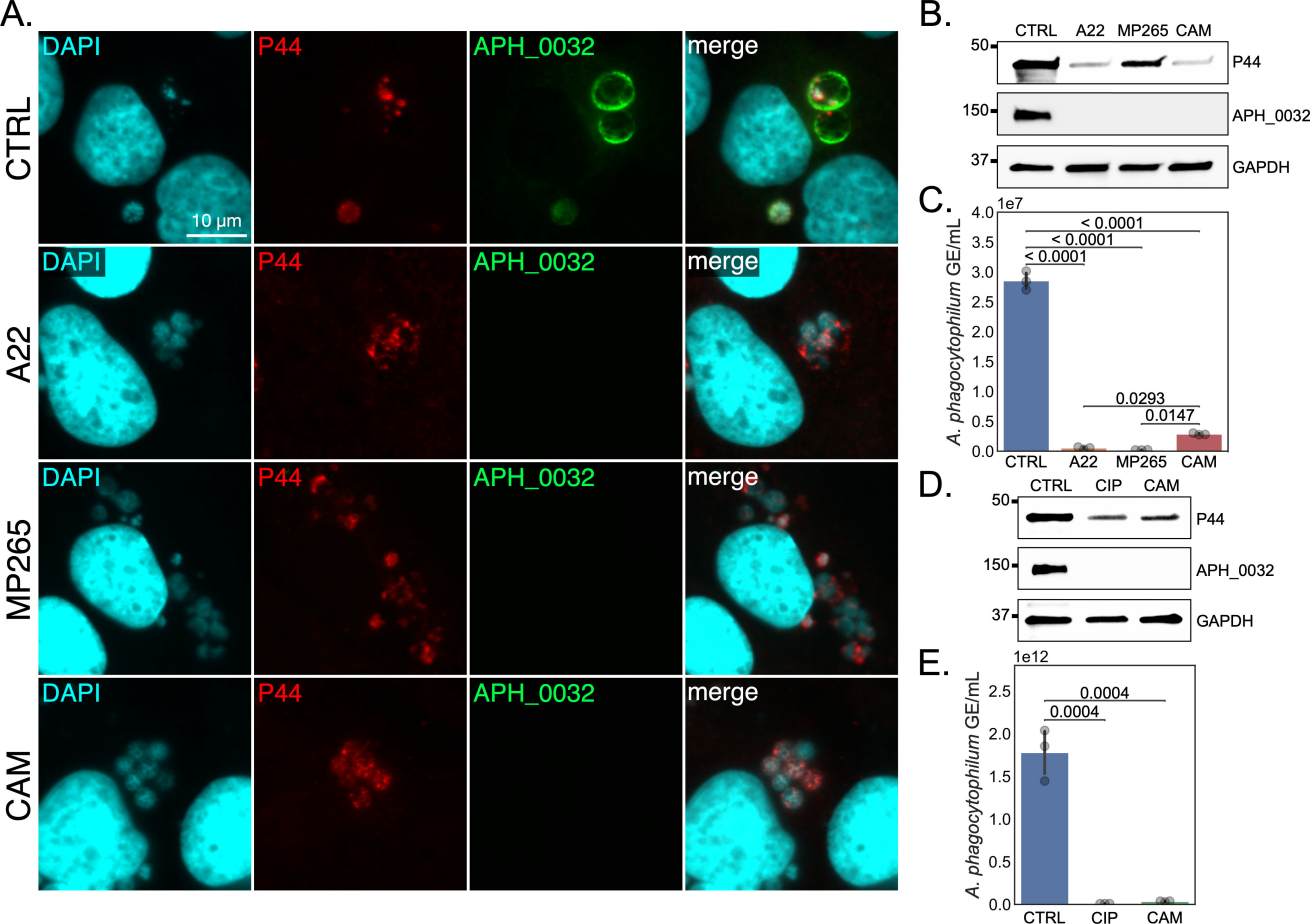
Cell division inhibition prevents ApV maturation and infectious progeny release. RF/6A cells synchronously infected with *A. phagocytophilum* were treated with vehicle (CTRL), A22, MP265, or chloramphenicol (CAM) at 16 hpi. At 26 hpi, the cells were either (**A**) fixed, stained with DAPI, immunolabeled for P44 and APH_0032, and examined by immunofluorescence microscopy. Alternatively, (**B**) cells were lysed followed western blot analyses assessing P44, APH_0032, and GAPDH protein levels or (**C**) host cell-free media was collected and bacteria released into the extracellular milieu was measured by qPCR. (**D**) and (**E**) Synchronously infected cells treated with CTRL, ciprofloxacin (CIP), or CAM at 16 hpi were either (**D**) lysed and western blot analyses performed or (**E**) media was collected and assessed for bacterial release by qPCR at 26 hpi. Data are means ± SD of three experimental replicates per condition. A one-way analysis of variance (ANOVA), followed by a Tukey’s multiple comparisons analysis, was performed for statistical analysis on (**C**) and (**E**). For (**C**), the ANOVA had degrees of freedom of 3, 8; the *F-*statistic equaled 897.8; and the *P*-value was less than 0.0001. For (**E**), the ANOVA had degrees of freedom of 2, 6; the *F*-statistic equaled 100.6; and the *P*-value was less than 0.0001. The adjusted *P*-values for (**C**) and (**E**) multiple comparisons analyses are presented on the graph.

*A. phagocytophilum* must complete its developmental cycle to regain infectivity before exocytic release so that it can properly disseminate (36, 38, 40). We hypothesized that inhibiting bacterial cell division, and thus RC-to-DC conversion, would halt ApV maturation and exocytosis. Infected RF/6A cells were treated at 16 hpi with A22, MP265, or chloramphenicol and analyzed at 26 hpi. A22 and MP265 prevented ApV maturation as APH_0032 expression was blocked, and the vacuoles remained filled with bacteria (Fig. 10A and B). GE analysis of the extracellular milieu confirmed that A22 and MP265 prevented *A. phagocytophilum* release to a greater extent than even chloramphenicol (Fig. 10C). Ciprofloxacin also nullified APH_0032 expression and DC release (Fig. 10D and E). These data establish that *A. phagocytophilum* septation and DNA replication are requisite for late-stage ApV maturation and release of infectious DC progeny.

## DISCUSSION

*A. phagocytophilum* is a pleomorphic bacterium that transitions between DC and RC forms, each of which performs distinct tasks that are essential for completing the developmental cycle and perpetuating infection. Analysis of *A. phagocytophilum* developmental cycle temporal dynamics revealed a monophasic RC replication phase that extends from approximately 4–17 hpi followed by DC production that initiates by 18 hpi and peaks at around 28 hpi. These findings build on our previous study by clarifying that DC production initiates 6 h earlier than initially reported (28). The observed large dynamic range in DC formation and release indicates that individual RCs within a host cell develop into DCs at different rates relative to each other. Live-cell time-lapse microscopy further evidenced that DC differentiation and ApV exocytosis are highly asynchronous, with individual ApVs exocytosing DCs as early as 18 hpi, while others contained RCs that were delayed in DC formation and/or ApV exocytosis beyond 32 hpi. This asynchronicity in ApV exocytosis may explain why *A. phagocytophilum*-infected neutrophils exhibit protracted (≥18 h) degranulation at the population level (64) and is likely an evolved strategy that maximizes the opportunity for extracellular DCs, which remain infectious for <1 h, to find new host cells.

RCs replicate exponentially between 4 and 22 hpi, which, together with our TEM studies, suggests that RC replication occurs by binary fission. This would not be surprising as *A. phagocytophilum* has maintained the cell division protein FtsZ, a tubulin homolog that localizes to the midcell during division (14). Yet, it is intriguing to consider how RCs regulate FtsZ midcell location given their amorphous structure. Furthermore, *A. phagocytophilum* is predicted to lack all known machineries for FtsZ midcell localization, including the MinCDE and ParABS systems as well as SlmA, which negatively regulate FtsZ polymerization, and the MatP/ZapAB system that positions FtsZ to the midcell (65–71). TEM analysis also showed almost exclusive segregation of RC and DC populations across ApVs. This observation, our single-cell evidence for asymmetric division in DC production, and the endospore-like subcellular architecture of DCs collectively suggest that DC formation likely occurs through an asymmetrical sacrificial division process resembling sporulation (41–43). Polarization of P44 and APH_1235 on DCs further supports a role for cell division in DC formation, as cell division can result in daughter cells exhibiting polarized protein positioning (14). Asymmetrical division has also been proposed to produce the chlamydial elementary body (EB), which is analogous to the *A. phagocytophilum* DC in that it is infectious and exhibits spore-like characteristics, including being non-replicative and containing an electron dense nucleoid. Yet, contrary to *A. phagocytophilum* DC formation, chlamydial EB production is non-sacrificial (18, 19, 21). Although endospore formation is largely restricted to Gram-positive bacteria in the phylum *Bacillota*, the Gram-negative non-pathogens, *Sporomusa ovata* and *Rhodobacter johrii*, and the vacuole-adapted pathogen, *Coxiella burnetii*, potentially undergo sporulation or an analogous process thereof (72–75). Whether bacteria outside the *Bacillota* truly sporulate remains contested. Indeed, even though *A. phagocytophilum* DC formation and DCs themselves superficially resemble sporulation and endospores, respectively, the bacterium is predicted to lack genes required for canonical endospore environmental resiliency (76, 77). Moreover, DCs markedly lose infectivity within 1 h of being extracellular in tissue culture media under normal culture conditions. Of note, chlamydial EBs are also labile when outside host cells (78).

Why *A. phagocytophilum* and *Chlamydia* spp. adopted pleomorphic lifestyles to create DC/EB morphotypes remains a mystery. Intriguingly, chlamydial infections are often persistent (79, 80), and it was recently shown that the EB can facilitate such persistence. Specifically, *Chlamydia trachomatis* remain in the EB form within host cells treated with the ciprofloxacin analog, ofloxacin, as well as chloramphenicol and tetracycline, and resumes its infection cycle upon drug removal (20). Members of the *Anaplasmataceae*, including *Anaplasma* and *Ehrlichia* spp., can establish persistent infections in animals (81–97). While this has been attributed to humoral immunity evasion through antigenic variation (81, 95, 96, 98), it has yet to be shown whether other mechanisms are involved. Notably, the bacteriostatic effect of desipramine, which inhibits eukaryotic acid sphingomyelinase to impede *A. phagocytophilum* sphingolipid parasitism and thereby halt the infection cycle, is reversed once treatment is stopped and the infection cycle progresses both *in vitro* and *in vivo* (38, 99). Assessing whether *Anaplasmataceae* DCs mediate persistence under antibiotic treatment or other stressors will be an important line of future research.

Our results establish MreB*^Ap^* as a *bona fide* cell division protein. MreB*^Ap^* localizes to the division plane of dividing RCs, and its inhibition produces aberrantly large *A. phagocytophilum* cells that are incapable of septation. Ciprofloxacin and chloramphenicol, which do not target MreB, also halt *A. phagocytophilum* replication but fail to induce aberrantly large cells. Furthermore, inhibiting septation with A22/MP265 or DNA replication with ciprofloxacin blocks RC-to-DC conversion, indicating that cell division is required for DC formation. While off-target effects of A22 and MP265 on *A. phagocytophilum* growth cannot be discounted, the facts that *A. phagocytophilum* biomass increases and protein expression is unaffected when MreB is inhibited suggest that this is unlikely. Intriguingly, the phenomenon of increased cell size and prevention of DC formation resembles that of chlamydial cell division inhibition by both beta-lactams and to a lesser extent A22/MP265 treatment (19, 54, 100). Additionally, the formation of aberrant *Chlamydia* via MreB inhibition has been shown to depend on septal peptidoglycan formation (54). However, *A. phagocytophilum* has jettisoned peptidoglycan coding genes from its genome and is resistant to beta-lactam treatment, suggesting that MreB inhibition prevents septation via alternative means perhaps by disrupting its interaction with FtsZ or by an analogous structural mechanism needed for septal plane formation (46, 101–103). Lastly, our data showed that cell division regulates expression of the late-stage ApV-associated effector, APH_0032, and subsequent ApV exocytosis, suggesting that cell division, commensurate with DC formation, controls multiple mechanisms involved in vacuolar maturation and bacterial dissemination.

The cues that govern *Anaplasmataceae* bacteria switching between morphotypes are unknown. The asynchronicity of RC-to-DC conversion across ApVs and the seemingly uniform shift of every RC to a DC within a given ApV suggest that morphotype conversion is controlled at the individual ApV level by a quorum sensing-like mechanism. However, *A. phagocytophilum* is predicted to lack canonical enzymes that produce quorum sensing molecules (104). Because 29% of the bacterium’s genome encodes hypothetical proteins, it cannot be ruled out that some of these regulate non-canonical quorum sensing processes that have yet to be defined (105). *A. phagocytophilum* is also predicted to encode at least three two-component systems (TCS) (104, 106), which could facilitate cell-to-cell communication within ApVs. Furthermore, the TCS protein CtrA is DC-specific in the closely related pathogen, *Ehrlichia chaffeensis* (107). Lastly, *A. phagocytophilum* contains two sigma factors, the constitutive σ^70^ and the alternative factor σ^32^ (104). Transcription of σ^32^ is increased during both *E. chaffeensis* entry and exit, suggesting its potentially involvement in RC-to-DC conversion (108). An analogous system exists in *C. trachomatis*, where its alternative sigma factor, σ^54^, controls much of the EB conversion regulon (109). Uncovering and validating the signal(s) and mechanism(s) of RC-to-DC differentiation may prove difficult, as genetic manipulation of this family of pathogens remains limited and morphotype conversion appears to be an essential process.

**Fig 11:**
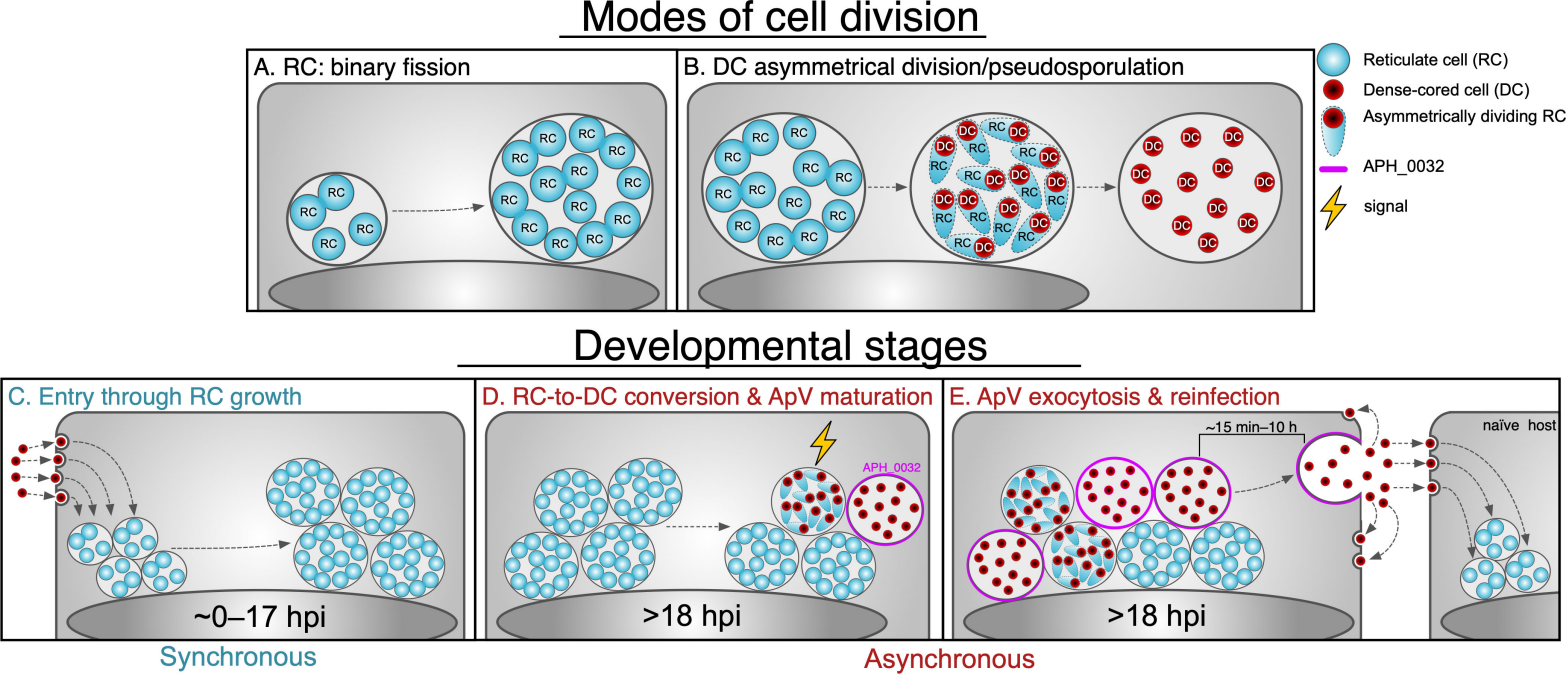
Schematic model delineating distinct stages of *A. phagocytophilum* development in mammalian host cells. (**A, B**) Modes of cell division. (**A**) RCs undergo binary fission to exponentially expand the bacterial population. (**B**) RC-to-DC conversion occurs through asymmetrical division that resembles sporulation and results in an exclusively DC population within the ApV. (**C**) to (**E**) Developmental stages. (**C**) In experimental infections, *A. phagocytophilum* entry, DC-to-RC transition, and RC replication and growth occur from 0–17 hpi. This portion of the developmental cycle is synchronous. (**D**) At ~18 hpi, individual ApV begin to receive an unknown signal that initiates RC-to-DC conversion and ApV maturation, as denoted by APH_0032 being expressed and localizing to the ApV membrane. This process occurs at the individual ApV level and is highly asynchronous. (**E**) ApV exocytosis and reinfection. Exocytosis of individual ApVs is in-of-itself highly asynchronous, occurring in as little as 15 min post-DC formation to >10 h later.

Overall, the data herein suggest that *A. phagocytophilum* employs differential modes of cell division during distinct stages of morphotype development, where RCs undergo binary fission to exponentially increase bacterial numbers and infectious DCs are formed through an asymmetrical, sacrificial division process (Fig. 11A and B). Additionally, we speculate that the act of asymmetrical DC production initiates a late-stage regulon that controls expression of bacterial factors to promote ApV maturation and exocytosis, and thus DC dissemination. The *A. phagocytophilum* developmental cycle also occurs in two phases: a synchronous phase consisting of a monophasic replicating RC population and an asynchronous phase where at ~18 hpi RCs within individual ApVs receive an unknown signal that triggers DC formation and ApV maturation (i.e., ApVs become APH_0032-positive) (Fig. 11C and D). This asynchronicity in development is exacerbated by the large variation in ApV exocytosis dynamics (Fig. 11E), which ensures that *A. phagocytophilum* exists in each morphotype concurrently and provides ever-present replicating and infectious populations.

This study focused on *A. phagocytophilum* development within a mammalian system. What the temporal dynamics of morphotype development are within the tick vector remain largely unexplored and will likely prove to be a fascinating avenue of future research. Finally, all other pathogenic members of the *Anaplasmataceae* exhibit disparate morphotypes (6, 110–113), hinting that pleomorphism may be conserved and thus regulated in a similar fashion in this family. As such, this study lays the groundwork for interrogating morphotype regulation across the numerous understudied bacteria in this family of emerging pathogens.

## MATERIALS AND METHODS

### Cell line and *A. phagocytophilum* cultivation

Human promyelocytic HL-60 cells (CCL-240; American Type Culture Collection [ATCC]) and *Macaca mulatta* RF/6A choroidal endothelial cells (CRL-1780; ATCC) were cultured as described (22, 114). *A. phagocytophilum* strain NCH-1 was grown continuously in HL-60 or RF/6 A cells in 75 cm^2^ flasks. For HL-60 cells, *A. phagocytophilum* was passaged as described (115). For RF/6A cell culture, 0.5–2.0 mL of media from an infected culture was transferred to a naïve confluent flask at 48 hpi. For infection experiments, DCs naturally released into the media were isolated via filtration through a two µm filter followed by sonication (5 s burst at 30 amps) using a S-4000 Ultrasonic Liquid Processor (Misonix).

### Synchronous infections

HL-60 or RF/6A cells were infected with DCs in Iscove’s modified Dulbecco’s medium (IMDM; Invitrogen) supplemented with 10% (vol/vol) heat-inactivated fetal bovine serum (hiFBS; Gemini Bioproducts) or in Dulbecco’s modified Eagle’s medium (DMEM; Gibco) supplemented with 10% (vol/vol) hiFBS; 1× Minimal Essential Medium containing non-essential amino acids (Gibco); and 20 mM HEPES (Gibco), respectively. Host cells and inocula were centrifuged at 1,000 × *g* for 3 min followed by incubation in a humidified incubator at 37°C with 5% atmospheric CO_2_ for 1 h. The inoculum was removed, and cells were washed twice with prewarmed (37°C) Hanks balanced salt solution and placed in fresh media. An MOI between 1 and 10 was used dependent on the experiment and confirmed via immunofluorescence or phase microscopy. For cell division experiments, at 16 hpi, the media was replaced with new prewarmed (37°C) media containing 50 µM of A22, 100 µM of MP265, 30 µM of ciprofloxacin, or 105 µM chloramphenicol.

### Live cell microscopy

RF/6A cells were grown to confluency in 24-well glass-bottom plates followed by synchronous infection with DCs. Cultures were grown in an Oko-Touch CO_2_-heated stage incubator at 37°C with 5% CO_2_. Qualitative developmental dynamic images were taken every 5 min from 16 to 35 hpi. For cell division inhibitor experiments, bacterial size and count were visualized by incubation with Hoechst 33342 (Invitrogen) at 1 µg mL^−1^ for 15–30 min in prewarmed media. Fluorescent and phase micrographs were acquired using a Leica DMi8 inverted epifluorescence microscope equipped with an Andor iXon Life 888 electron-multiplying charge-coupled device (EMCCD) camera (Oxford Instruments) and EL6000. A 40×/0.6 numerical aperture (NA) dry or 100×/1.3 NA oil immersion objective lens was alternatively used, dependent on experiment. Bacterial size and count were measured per ApV.

### P44 antiserum generation

*A. phagocytophilum* Dog2 (GenBank AGR822228.1; locus tag YYY_05880) nucleotide sequence encoding residues 22–395 was synthesized and cloned into pUC57 by Genewiz. The coding sequence was subcloned into pET-46 Ek/LIC (Novagen) downstream and in-frame with a 6xHis tag coding sequence according to the manufacturer’s instructions. The plasmid was transformed into *E. coli* Stellar Competent Cells (TaKaRa Bio), and 6×His-P44 was purified from the insoluble phase as described previously (116). The purified protein was inoculated into Sprague-Dawley rats, yielding rat anti-P44 antiserum, following previously described protocols (117).

### Immunofluorescence microscopy

For cell division inhibition experiments, RF/6A cells were grown to confluency on 12 mm glass coverslips (Bellco Glass) in polystyrene 24-well plates, followed by synchronous infection with DCs. For MreB localization experiments, liberated RCs were centrifuged onto microscope slides using a Cytospin 4 (ThermoFisher Scientific) at 1,000 × *g* for 3 min. For MreB immunolabeling, infections were fixed with ice-cold methanol for 10 min. For APH_0032, APH_1235, and vimentin immunolabeling, samples were incubated in 4% paraformaldehyde (PFA) (32%; Electron Microscopy Sciences) diluted in 1× phosphate-buffered saline (PBS) at room temperature for 20 min, followed by treatment with 0.1% Triton-X 100 (Fisher Scientific) for 10 min. Samples were rinsed thrice in 1× PBS, and incubated in primary antibody for one h in 1× PBS. Primary antisera and antibodies used in this study were APH_0032 rat antiserum (1:1,000) (22), APH_1235 rat antiserum (1:1,000) (22), P44 rabbit antiserum (1:1,000) (22), P44 rat antiserum (1:1,000), anti-vimentin (1:1,000 [AB8069; Abcam]), and anti-MreB (1:200 [PA5-144554; Invitrogen]). Samples were rinsed thrice in 1X PBS and incubated for 1 h with Alexa Fluor 488- or 594-conjugated chicken anti-rabbit IgG, Alexa Fluor 488- or 594-conjugated chicken anti-rabbit IgG, or Alexa Fluor 700-conjugated goat anti-rabbit IgG (ThermoFisher Scientific) at a 1:1,000 dilution in 1× PBS together with 0.1 µg mL^−1^ DAPI (ThermoFisher Scientific). Three final rinses were performed and coverslips mounted with Prolong Gold Anti-Fade reagent (ThermoFisher Scientific). ApV quantification micrographs were acquired with a Leica DMi8 inverted laser-scanning confocal microscope (Leica Microsystems) equipped with a Stellaris8/Falcon system and white-light laser. A 63×/1.4 NA oil immersion objective lens was used. For assessing APH_1235 polarization, MreB subcellular localization, and cell division inhibition experiments, the same Leica DMi8 from the live cell experiments was used with an 100×/1.2NA oil immersion objective lens.

### Transmission electron microscopy

For visualization of *A. phagocytophilum* cell division and DC ultrastructure, ~2.0 × 10^5^ RF/6A cells were seeded onto 60 × 15 mm Permanox dishes (Electron Microscopy Sciences), followed by infection with *A. phagocytophilum* DC organisms. Samples were prepped and imaged as previously described (22). Grids were visualized using a JEOL JEM-14000Plus transmission electron microscope (JEOL) with the Gatan OneView 4K × 4K direct electron CMOS camera (Gatan). For Immunoelectron microscopy ~9.0 × 10^6^ *A. phagocytophilum-*infected HL-60 cells were used. Samples were prepped as previously described (28). The grids were washed twice with 1× PBS, blocked with 0.1% bovine serum albumin in 1× PBS for 1 h, and immunolabeled with either rat APH_1235 antiserum or mouse P44 monoclonal antibody 20B4 (118, 119) at a 1:10 dilution conjugated to 6 nm gold particles (Electron Microscopy Sciences). Grids were imaged using a JEM-1230 transmission electron microscope (JOEL) equipped with a Gatan UltraScan 4000SP 4K × 4K CCD camera (Gatan).

### Reinfection assays

Unless otherwise stated, DCs were pelleted from the media of infected RF/6A cells by centrifugation at 20,000 × *g* for 20 min at 4°C. Pellets were resuspended in DMEM via brief sonication to homogenize the DCs and rupture any host cells. For extracellular viability, infectious media was processed through a 2 µm filter to remove host debris. For reinfection, RF/6A cells were seeded to confluency in 96-well microtiter plates. RF/6A cells were synchronously infected with infectious media via two-fold serial dilutions across the 96-well plate in 100 µL total volume. Infected RF/6A cell media was supplemented with chloramphenicol (105 µM) at 16 hpi. Cells were fixed at 24 hpi with ice-cold methanol for 10 min and stained with DAPI and immunolabeled with P44 rabbit antiserum followed by secondary antibody labeling with Alexa Fluor 594-conjugated anti-rabbit IgG chicken antibody (1:1,000 [ThermoFisher Scientific]). Labeled cells were imaged via automated microscopy using a Leica DMi8 epifluorescence microscope with a 40×/0.8NA dry objective lens and the Leica Microsystems LAS-X 3.7.4 software. Automated focusing was performed on the DAPI-stained host nuclei. Two dilutions series (technical replicates) were performed per experimental replicate. The mean of four FOVs per dilution was calculated to determine the mean of each technical replicate. The infectious titer was back calculated based on dilution and well surface area. ApV numbers were measured in wells containing less than 50 ApVs per FOV.

### GE quantification

*A. phagocytophilum*-infected RF/6A cells or released *A. phagocytophilum* DCs were pelleted by centrifugation at 20,000 × *g* for 20 min at 4°C, and the supernatants were removed. Pellets were frozen at −20°C until processing. DNA was purified using the DNeasy Kit (Qiagen). *A. phagocytophilum* GE were quantified via detection of a 151 bp region of the *A. phagocytophilum* NCH-1 *dnaK* gene with primers 5′-acatgagggatttgaagtgtccttatgagg-3′ and 5′-gcaccaaagtatctctcagcagtttcc-3′ using SsoFast EvaGreen Supermix, a CFX96 Real Time PCR Detection System, and CFX Maestro software (Bio-Rad Laboratories). *A. phagocytophilum* GEs were determined from a standard curve using the pCR4-TOPO TA Vector (Invitrogen) containing the *dnaK* ORF (Accession: KJV68131; GeneID: EPHNCH_0581). Standard plasmid construction was performed as follows. *A. phagocytophilum* genomic DNA was purified from naturally released DCs. *dnaK* was amplified using the primers 5′-atggcggctgagcgtataatagg-3′ and 5′-ctaagtattcttcttgtcctcgtccttattaatctcc-3′. The amplicon was inserted into pCR4-TOPO TA per the manufacturer’s instructions and transformed into chemically competent *E. coli* NEB 5-alpha cells (New England Biolabs). Insertion sequence was confirmed via Sanger sequencing (Azenta).

### Western blot and densitometric analysis

For *A. phagocytophilum* developmental cycle experiments, infected monolayers and media or monolayers only were harvested and pelleted by centrifugation at 20,000 × *g* for 20 min at 4°C. Samples were lysed and processed as previously described (117). Nitrocellulose membranes were probed overnight at 4°C for GAPDH mouse monoclonal antibody (1:1,000 [sc-365062; Santa Cruz]), APH_0032 rat antiserum (1:1,000), APH_1235 rat antiserum (1:500), or P44 rabbit antiserum (1:1,000) in Tris-buffered saline plus 0.05% (vol/vol) Tween-20, containing 5% (vol/vol) nonfat dry milk. Secondary antibodies were horseradish peroxidase-conjugated to anti-mouse IgG, anti-rat IgG, or anti-rabbit IgG (1:10,000 [Cell Signaling Technology]) and were incubated for 1 h at room temperature. All blots were incubated with SuperSignal West Pico PLUS, SuperSignal West Dura PLUS, or SuperSignal West Femto PLUS chemiluminescent substrates (ThermoFisher Scientific) per manufacturer’s instructions. Blots were imaged using ChemiDoc Touch Imaging System (Bio-Rad) and processed with Bio-Rad Image Lab 6.0 software to obtain densitometric values.

### Statistical analyses and data presentation

Statistical analyses were performed using the Prism 7.0 software package (GraphPad). For experiments comparing two groups, independent *t*-tests were performed and *P*-values reported. For experiments with greater than two groups, one-way analysis of variance (ANOVA), followed by a Tukey’s multiple comparisons analysis was performed. Degrees of freedom, *F*-statistics, and *P*-values for ANOVAs are reported in figure legends. Adjusted *P*-values for the multiple comparisons analyses are reported within the figure itself. Alpha was set to 0.05 for all statistical analyses. Linear regressions were performed using the Numbers application (Apple). Micrographs were processed and analyzed using Fiji (120), (version 2.16.0/1.54 g). Data were visualized with Pandas, Matplotlib, and Seaborn using custom Python scripts in Jupyter Notebook (LF Charities). Figures were assembled in the Graphic application (Picta Inc.).

## Data Availability

All data, bacterial strains, and methodologies are available upon request.

## SUPPLEMENTAL MATERIAL

Legend mbio.01972-25-s0001.docx Legend for the 4 supplemental movies.

Movie S1 mbio.01972-25-s0002.avi RF/6A cells synchronously infected with *A. phagocytophilum.*

Movie S2 mbio.01972-25-s0003.avi RF/6A cells synchronously infected with *A. phagocytophilum*.

Movie S3 mbio.01972-25-s0004.avi RF/6A cells synchronously infected with *A. phagocytophilum*.

Movie S4 mbio.01972-25-s0005.avi RF/6A cells synchronously infected with *A. phagocytophilum*.

ASM does not own the copyrights to Supplemental Material that may be linked to, or accessed through, an article. The authors have granted ASM a non-exclusive, world-wide license to publish the Supplemental Material files. Please contact the corresponding author directly for reuse.
