# Supplementary material for: Distinct modes of cell division drive Anaplasma phagocytophilum morphotype development and the infection cycle
Source: mBio. 2025 Aug 25;16(10):e01972-25. doi: 10.1128/mbio.01972-25 (PMC12506133; doi:10.1128/mbio.01972-25)
Supplement: Legend — for the 4 supplemental movies. [file mbio.01972-25-s0001.docx]

**SUPPLEMENTAL MATERIAL**

**Movies S1-4.** Live-cell time-lapse movies of RF/6A cells synchronously infected with *A. phagocytophilum*. Images were taken every 5 min from 16 to 35 hpi using transmitted phase light at 40X magnification.
